# Some Impressions of Certain Sessions of the National Dental Association*Reprinted from the *Pacific Dental Gazette* for January, 1918.

**Published:** 1918-02

**Authors:** B. Frank Gray

**Affiliations:** Colorado Springs


### SOME IMPRESSIONS OF CERTAIN SESSIONS OF THE NATIONAL DENTAL ASSOCIATION *

BY DR. B. FRANK GRAY, COLORADO SPRINGS

I shall merely attempt to give a birdseye view or impression of some of the sessions of the great meeting of the N. D. A. recently held in New York City. (Other members of the Los Angeles County Dental Society had the privilege of attendance, and I have no doubt that they will in due time cover more specifically certain interesting phases of the meeting.)

At all of these large conventions which I have at-

* Reprinted from the *Pacific Dental Gazette* for January, 1918.

tended I hear occasionally the complaint that the meeting is so large that it is unwieldy and not altogether well managed. I think that is true in a certain sense. But I also believe that if we stop a moment to consider the situation we will appreciate that with the tremendous annual growth of the N. D. A. it is only a wonder that the sessions may be carried on with even a tolerable degree of success. The membership of the N. D. A. was about 2,500 only a very few years ago. What it is now I am not sure—probably 20,000 or considerably more. There was a registration in New York of 5,000 dentists, approximately.

Granting that such a meeting were handled in the best possible manner, what dentist is there among us who could assimilate all that was covered by the program, even though he could attend every single session and every single clinic? I fell that the man who is content to learn what he can, realizing that it is but a small part of the whole, is the one who is in a proper frame of mind to profit most by these great conventions.

Doubtless all of you have looked the program over, and have a general impression of the manner in which the meeting was conducted. The clinics, so far as I know, were of the "continuous performance" type, which is to say that there might be seven or eight clinicians working on different subjects in the same room, occupying separate booths. Fifteen or twenty dentists were admitted to each booth. The operator started his demonstration on the stroke of a bell and finished at a given signal. Then all present would move forward to the next clinician's booth. In this way all of the clinics can be seen in a very comprehensive manner and in a comparatively short period of time.

On the morning of October 22, at the first general session, Dr. E. C. Rosenow, of the Mayo Foundation, Rochester, Minn., gave an interesting address. A man so much quoted and whose work is being so closely watched needs a bit of characterization. If Dr. Rosenow is anything he is sincere and speaks with the greatest possible degree of modesty. His manner is thoughtful and kindly. One of his slogans was, "Clean the mouth first!" "Frequently a dying infected pulp occurring in a normal tooth is the source of a hematogenous infection. That spells focal infection, and the moment it occurs you and the medical profession are on common ground. Clean the mouth first!" Dr. Rosenow stated that the tonsils might be removed and with infected teeth left in the mouth the patient might be even worse than before. Some teeth can be devitalized and the root canals properly filled. Certainly extractions should not be done in a wholesale manner. The removal of bad teeth may be followed by relief or cure of infected tonsils. The tonsils is the line of defense and there is where the infection goes. He spoke of a patient who was suffering from ulcer of the stomach. Since the physician found pus about the tonsils, he removed them. The patient grew better and then returned to the same condition as before. This time Dr. Rosenow found pus around three molar teeth. These teeth were removed and the patient promptly recovered and has had no recurrence of the trouble. So the doctor says, "We have to be sure of eliminating the focus of infection." The focus may be extremely small and yet cause grave troubles. He cautioned the members present against making flattering promises to patients suffering from diseases due to focal infections. Finally he emphasized his belief that the two professions should be united in this affair.

On October 23, 8 p. m., Dr. Bunting, of the Research Department of the N. D. A., gave quite an extended report of his work on dental caries. He had much to say on the laws of osmosis as they apply to the dentin, enamel and cementum. He finds that certain substances may act as a permeable membrane. For instance, salt solutions will pass through enamel and dentin in a test tube, and he therefore contends that in the mouth the salts of the blood inside and of the saliva outside will have a similar osmotic flow. He feels that this opens a wide field of speculation as to many dental phenomena that have not as yet been explained. The change in quality and appearance of the teeth corresponding to the different periods in life may be explained by the change of osmotic flow, etc. Thus he felt that this principle of osmosis is related to caries of the teeth. In like manner the discoloration of teeth after devitalization may be explained by osmosis. The same he felt to be true of hyperesthesia. Teeth become sensitive after vigorous prophylactic treatment; previous to this time the teeth may have been covered with a more or less colloidal substance or film; then osmotic interchange of fluids may take place (after the prophylactic attention) and sensitivity will ensue for a time. He stated in conclusion that he was convinced the enamel and dentin of the teeth are more or less porous and will admit of the passage of salts, etc., from the blood outward or from the saliva inward. This interchange takes place in a variable manner, due to the changes in the composition of the blood and of the saliva.

On the same evening Dr. Thomas B. Hartzell, of the Research Foundation, stated: ''The address of Dr. Rosenow has complimented in an able way the knowledge which we have been presenting to you for the last three or four years.'' Dr. Hartzell said the relation of secondary infections to primary infections in the mouth had been treated very meagerly previous to the work which was commenced in Minnesota three or four years ago, and that the question of what we shall do with these types of infection is the biggest thing in dentistry today.

Dr. Weston A. Price, of the Research Institute, is probably the most indefatigable worker in the profession today. He seems to be a veritable steam engine. I remember addressing a letter to him about two years ago, asking him what to do with teeth which the radiograph showed to have imperfectly filled root canals, although no area of rarefaction was in evidence; or what would he do with those teeth which showed very small areas about their roots. I have stated before this society on another occasion his response, in effect that those were the very problems they were working on in the Research Institute. Without wishing to be quoted at that time, he said that since we do not know how much infection is enough to cause serious disturbances, it was not absolutely safe to leave any infection present in or about the teeth. Under Dr. Price's direction much experimental work has been performed in Cleveland during the past year along these lines. Certain methods of root sterilization have been worked out that should prove of immense interest to the profession. I certainly hope all our members will study carefully the reports from the Research Institute as they are published from time to time. One comment by Dr. Price with reference to the sterilization of tissues with the electric current (ionization) was, "You are spending almost three-quarters if not nine-tenths of your efforts on the vital tissues instead of on the pathological tissues simply because the current takes the path of least resistance." I do not believe this needs any comment.

The experiments in the Cleveland laboratories with different sterilizing agents are certainly very interesting. It seems to have been the practice of the profession through the years to allow various sterilizing agents or medicaments to remain in infected teeth for rather long periods of time, in the belief that the efficiency of the agent was increased by the length of time the agent was left in place. I am sure you will be interested in looking up the experiments which have caused Dr. Price to conclude that this practice is all wrong. In a general way he now concludes as a result of these experiments that the briefer the time (within reason) a drug is allowed to remain in the tooth, the greater will be its sterilizing efficiency. He said, therefore, ''Make short treatments. How short? Five minutes or five hours—must be worked out. I think a fifteen-minute treatment is going to be much more efficient than a fifteen-hour treatment.''

Referring again to the matter of when to extract teeth and when not to, I gathered from Dr. Price's various statements that he has concluded that this whole question is one of resistance. One individual may have teeth which are seriously infected and yet suffer few, if any, bad consequences. Another person with a small focus of infection may be thereby subjected to serious disease. One patient has a susceptibility to a certain kind of disease, and it is the duty of the dentist to study his patient. Teeth that should by all means be extracted for one individual might be retained in the case of another patient.

Major William H. G. Logan, of the Army Medical Corps, spoke at the third general session on the evening of October 24. We all have in mind the recent request of the N. D. A. to use our influence with reference to certain important legislation affecting the dental corps of the army. As we know, this important service has not had a fair status or rank. While in Ann Arbor recently I was told by a dentist, whose name is familiar to all of you, that a very prominent medical man who exerted a most wonderful influence in Washington, used that influence directly against the army dental bill. But what happened? Almost as though by magic, after the influence of the profession was brought to bear throughout the country, the desired legislation went through with a whirl. Major Logan has received credit in certain quarters for the passage of the amendment to the dental law. However, the Major stated most emphatically that he was not the man who exerted the largest influence. Of course he worked for the desired legislation, but the greatest credit was due to Emery C. Bryant and Homer C. Brown, of Washington, D. C., and Columbus, Ohio, respectively. Major Logan entered the Medical Department of the Army in preference to the dental branch for the reason that he believed he could do the most good for dentistry in the medical service. When his fellow medical officers questioned him with reference to the merit of the dental measures desired, it seems they did not quite realize that he, while a physician, was first and foremost a dentist. You may readily understand the wonderful power of his influence in just this way. I understood Major Logan to say that for the present they have all the dentists needed for the service. However, with further drafts I presume the demand for operators will still be present.

The work of John R. Callahan, of Cincinnati, is pretty well known to the profession. He made a report in the Research Department on October 24. However, he did not treat of his work in root canal technic at this time. He showed on the screen a number of pictures about which he made very few comments, preferring, as he said, merely to have those present see the slides and start them thinking a bit. As an example, he would show a slide made from a section of the mandible showing the root end and its very close proximity to the inferior dental canal. He also called attention to the fact that the interior of the bone was much of the nature of marrow. The inference given was that an intelligent operator would scarcely want to force dangerous and corroding medicines through the apex of a tooth into structures of the character he was picturing. Another point. I believe the average dentist in the case of a tooth showing a decided area at the apex feels that its extraction is all that is necessary. Dr. Callahan stated in the case of a tooth extracted twenty-three years previously an X-ray picture showed a rarefied condition. A curettment was made; a culture was secured showing the diplococcus. This patient had been incapacitated, and after proper treatment went back to his work within a few weeks.

Along the line of root sterilization I want merely to call attention to the work of Percy R. Howe done in, and with the assistance of, the Forsyth Dental Infirmary. Briefly, it has to do with the treatment of teeth with a preparation of silver. The metal is caused to actually penetrate the dentinal tubuli. The efficiency of the method is most highly regarded, I believe, by the Research Department. About the only objection to this method which was cited was its tendency to seriously discolor teeth—especially in the front of the mouth. However, certain means of overcoming this difficulty were suggested. I seriously advise you all to look up the data on this subject, for I believe it will have a very splendid application in your every-day work.

In Section I on Tuesday, October 23, a paper was read by Dr. W. L. Fickes, of Pittsburgh, entitled "The Porcelain Inlay: Its Strength and Durability." This paper, with its discussion, served to emphasize the fact that now as in the days of its more common use, the porcelain inlay when made by an artist in that particular class of work, probably has a place in dentistry which can not really be supplied by any other material.

Another paper presented in this section on the same day provoked a rather heated discussion in the matter of the gold inlay vs. the malleted foil filling. Dr. Prime, of Omaha, contended that if we succeed in performing a good operation, whether it be in amalgam, in the cast gold inlay, in the porcelain inlay, or in the malleted gold filling, success comes only at the price of eternal vigilance—that a man who can make a good cast inlay will make a good foil operation, and the man making a good foil operation can learn to make a good porcelain inlay. Only the careful, the studious, the honest, and the skillful will succeed in either. Dr. Prime was glad to hear mentioned the excellency of the porcelain inlay. Where indicated, he said, it is the peer of all. Likewise he was hoping to hear some of those present say that the gold foil filling was still favored by them, saying, "the time and skill employed to produce a good gold inlay will produce in the same man's hands a better operation in gold foil in the majority of cases."

The discussion of a paper by Dr. Fred E. Hart, of San Francisco (Some Neglected Operative Perquisites), brought out some interesting discussion. Dr. Gethro, of Chicago, stated, "We are having trouble with inlays throughout the country; we are finding margins that are bad now which were fairly good when they left the office. Many teeth are being split." The cause of this he felt was that the material used is not stationary—the pure gold will not stand the stress of occlusion, if it is normal, for a great length of time without change in form. He said that a gold inlay of 24 carat can not be compared with a good foil filling. Neither may 24 carat be compared with hammered gold of the same fineness. He felt that an inlay of pure gold is almost worthless in certain positions. However, he recognized the advantage of getting better margins with the pure gold and would favor its use in positions where there is no stress. Again he felt that it was a mistake to allow the patient to bite into the wax for securing the occlusion. He said that it would distort some of the wax away from the margins. He prefers to carve the wax up. The contact point he would always make of solder, no investment being necessary.

In discussing the last paper mentioned, Dr. Prime had much to say about the use of the rubber dam. He said that if he were to make an implantation of infected material how better would he do it than by going to the automobile shop and securing a piece of inner tubing made at the Goodrich Company, punch a hole in it, pay no attention to the infective materials on the surface of the tooth—push the rubber down over it, carrying the infection with it; and then to make doubly sure of the thing, he would take a ligature and tear the fibres of the peridental membrane or traumatize them, then to be still more sure, he would tie the ligature around it and hold the infection in the lesion for two hours! And when we lift up the piece of tubing we discover the tissues all blanched—we have driven from the field the only possible agent nature has to combat the infection. He said that one could not beat this method with a hypodermic. He stated that of course we had to use the rubber dam, but we should use it intelligently, carefully and thoughtfully, using no ligatures unless actually forced to do so. The same was true of clamps.

C. C. Voght, A.B., Ph.B., of the Mellon Institute of Industrial Research in Pittsburgh, gave a most illuminating paper on the subject of "The Chemistry of Silicates and Their Application in Dentistry." According to the opinions of those discussing this paper, one of the greatest mistakes in the use of silicate cements was the polishing or finishing of the filling too soon after its insertion. This work should not be done for twenty-four hours after the filling is placed in the tooth. Dr. W. V. B. Ames, the veteran cement manufacturer, felt the subject of silicate cements had been seriously befogged by the names given these materials and the attempt to convey the idea that they are distinctly different from the oxyphosphates we have been using—that is, as to their chemistry. Up to the point of the secondary setting of these cements we have an oxyphosphate cement pure and simple, governed by the laws governing any other oxyphosphate we have been using. Dr. Ames felt that unlimited mixing and spatulation might be a disadvantage; in fact, over-spatulation might kill the result we are striving to get.

Dr. McKay's report on the "Progress of the Investigation of Mottled Enamel" was presented in the Research Department on Thursday morning, October 25. Quite a large and appreciative audience listened to his most interesting presentation. This report should be read by every member of the Colorado Springs Dental Society. You will not find it half so prosy as you might fear. I happen to know that it is intensely interesting.

Following Dr. McKay, William J. Gies, M.D., Ph.D., working in and with the assistance of Columbia University, gave an unusual verbal report of "Studies of Internal Secretions in Their Relation to the Development and Condition of the Teeth."

Dr. Gies carried on most interesting experiments with rats, rabbits and dogs. He found the removal of the thyroid, together with the para-thyroid, did not produce any noticeable effect on the teeth. Then the para-thyriods were removed and the thyroids left, and the result was impairment of calcification. Thus he believed the thyroid and the para-thyroid have to do with "balancing effects in the body." The connection of the para-thyroid glands with calcification is supported by Erdheim in his histological studies. Dr. Gies' studies from the "chemical side" support his observations.

Then the idea was conceived to add to the normal supply and note the effect on calcification of the teeth. Therefore thyroid was fed, as well as para-thyroid, salivary gland and lymphatic gland, and the calcification of the teeth was noted to be ''more than normally fine—greater in degree and more perfect in character.''

One of the interesting experiments was the injection of tripan blue into dogs—into pups at the nursing stage and before the formation of the second teeth, and into others after the teeth were almost fully formed. When these injections were made before the teeth were fully formed, the teeth took on a permanently blue color. Per contra, when the injection of the tripan blue was made into dogs *with fully formed teeth* the blue did not diffuse into the teeth. However, in a dog that was exhibited of the latter description, the gums, ears, the whole body (the animal was normally white) showed the blue most vividly—the teeth alone remaining perfectly white. The dog into which the injections were made *during the formation of the teeth* certainly presented wonderful blue teeth. As Dr. Gies said, ''Red gums, blue teeth and white eyes, a new fashion in teeth.''

Dr. Gies' report was most comprehensive. Its bearing on dentistry is obvious. The possibility of the diffusion of various substances into the teeth, during the formative period, has an intimate relation, it would appear, to the subject of mottled enamel and the brown stain which is being so exhaustively studied by Dr. Frederick S. McKay, of the Research Institute of the National Dental Association. Dr. McKay's recent report before the National Dental Association should be read by all progressive dentists.

In this ''running talk'' I have endeavored to give some impressions of those sessions of the great New York meeting I was privileged to attend. The inspiration and help of these annual meetings of our great central organization is certainly worth every effort we may make to attend them.

